# 宁养肺癌患者生存状况及其影响因素研究

**DOI:** 10.3779/j.issn.1009-3419.2012.07.04

**Published:** 2012-07-20

**Authors:** 皓 刘, 海强 郭, 玉梅 王, 嵘 刘, 雷雷 褚, 明 崔

**Affiliations:** 1 110001 沈阳，中国医科大学公共卫生学院卫生统计学教研室 Department of Health Statistics, School of Public Health, China Medical University, Shenyang 110001, China; 2 110022 沈阳，中国医科大学附属盛京医院宁养病房 Ward of Hospice Service, Shengjing Hospital, China Medical University, Shenyang 110022, China

**Keywords:** 肺肿瘤, 生存分析, 生活质量, 生存期, 影响因素, Lung neoplasms, Survival analysis, QOL, Survival time, Influencing factor

## Abstract

**背景与目的:**

生存期和生活质量结合能更全面地评价患者的生存状况。本研究旨在分析宁养肺癌患者的生存期与生活质量，探讨二者的影响因素，为改善患者生存状况提供依据。

**方法:**

对盛京医院宁养病房收治的晚期肺癌患者进行回顾分析及随访，采用SPSS 13.0软件处理数据，用*Kaplan-Meier*法计算中位生存期，用肿瘤患者生活质量量表评价患者生活质量，研究两者与各因素间的关系。

**结果:**

269例患者生活质量平均得分28.76分，中位生存期为10个月。患者对病情的了解、止痛效果、KPS评分和营养状况是生活质量的独立影响因素；手术、疼痛出现早晚、止痛效果和KPS评分是生存期的独立影响因素。

**结论:**

晚期肺癌患者生存状况较差，应从上述因素加强和完善宁养服务，提高患者生活质量并延长其生存期。

## Abstract (en)

**Background and objective:**

Survival and the quality of life more comprehensively evaluates the survival conditions of cancer patients. This paper aims to analyze the survival time and quality of life (QOL) of hospice lung cancer patients and discusses the influencing factors to provide a basis for ameliorating their survival conditions.

**Methods:**

Advanced lung cancer patients who are receiving hospice care in the Shengjing Hospital were retrospectively analyzed and followed-up. The data were analyzed using SPSS 13.0. The median survival of patients was determined using the *Kaplan-Meier* method, and their QOL were evaluated using the tumor patient's QOL scale. Finally, the relationship between the survival index and the various factors were determined.

**Results:**

For 269 patients, the average QOL score was 28.76 and the median survival was 10 months. Awareness of their illness, pain relief, Karnofsky performance status (KPS) score, and nutritional status are the independent factors of QOL. Surgery, time of the occurrence of pain, pain relief, and KPS score are the independent factors of survival time.

**Conclusions:**

The survival of patients with advanced cancer is poor; hence, we should strengthen and improve hospice care in terms of the influencing factors to enhance the QOL of patients and prolong their survival.

肺癌是世界上最常见的癌症，每年有120万新发病例^[1]^。WHO 2003年公布的资料^[2]^显示，肺癌无论是发病率还是死亡率均居全球癌症的首位。肺癌在我国也是最常见的恶性肿瘤之一，30多年来我国肺癌的发病率和死亡率呈现持续上升的趋势，已上升为城市的第1位死亡原因^[3]^。肺癌的治疗至今仍无突破性进展，约65%的肺癌患者确诊时已属晚期，预后甚差，约80%的患者在确诊后1年内死亡。肺癌治疗主要倾向于姑息性治疗，如何在延长患者生存期的同时提高患者生活质量，已成为日益受到重视的问题^[4, 5]^。本研究从生存期/生存率和生活质量两方面探讨接受宁养服务的晚期肺癌患者相关情况并分析其影响因素，旨在更全面地概括和反映患者的生存状况，为临床防治提供参考依据。

## 1 资料与方法

### 1.1 资料来源

研究对象来源于中国医科大学附属盛京医院宁养病房2007年1月-2009年12月登记的晚期肺癌患者共269例，均经病理检查证实或经临床、影像诊断且确诊日期明确。宁养院收治的居家宁养患者均为经济条件贫困持有低保证的患者，宁养院主要是定期向其发放一些止痛药物，如吗啡等，并给予一定的护理指导以及临终关怀。

### 1.2 调查方法

所有肺癌患者依据其病案资料了解其个人特征，明确诊断，回顾确诊以来的情况，并通过电话、入户进行生存随访，随访日期截止到2010年12月31日。随访的终点事件为因所患癌症死亡，生存期为确诊时间至因所患癌症死亡时间或随访终止时间。非因所患癌症死亡、失访病例及随访截止时仍存活病例按截尾数据分析处理。

### 1.3 调查工具

① 一般特征量表，其中含有患者的基本情况和生存分析需要的相关时间及事件条目等；②患者疼痛程度的评估方法采用0-10数字强度分级法（numerical rating scale, NRS）记录，0代表无痛，1-3为轻度疼痛，4-6为中度疼痛，7-10为重度疼痛；③Karnofsky performance status（KPS）评分表，KPS评分法是根据患者一般状况进行分级评分，最高100分，最低0分，每10分为1个等级，如100分表示一切正常，无不适或病征；90分表示能进行正常活动，有轻微病征；50分表示需要别人更多的帮助，并经常需要医疗护理；10分表示病情垂危；0分表示死亡。分数越低说明患者的一般状况越差^[6]^；④生活质量评价采用孙燕院士等^[7]^提出的肿瘤患者生活质量评分标准，生活质量（quality of life, QOL）由12项指标体现，包括食欲、睡眠、疼痛、日常生活状况、面部表情、治疗不良反应、疲劳、精神、自身对癌症的认识、对治疗的态度、家庭及同事的理解与配合。每项计1分-5分，生活质量总分为60分。得分≤20分为生活质量极差，20分 < 得分≤30分为差，30分 < 得分≤40分为一般，40分 < 得分≤50分为较好，50分 < 得分≤60分为良好。单项指标中≤2分者为该项质量差。以上各量表均被多次实践使用，信度和效度良好，调查由专人负责，按测评表逐项填写。

### 1.4 统计分析

采用SPSS 13.0软件包进行数据整理和统计学分析，采用乘积限法（*Kaplan-Meier*法）计算生存率和生存期，生存期单因素分析采用*Log-rank*检验，多因素分析采用*Cox*比例风险回归模型分析。生活质量的单因素分析采用卡方检验、秩和检验等，多因素分析采用*Logistic*回归。计量资料用均数表示，计数资料用例数（构成比）表示。检验水准取*α*=0.05。

## 2 结果

### 2.1 人口学特征

269例患者中男性165例（61.3%），女性104例（38.7%），男女性别比约为1.6:1。确诊时的年龄为23岁-95岁，平均60.29岁，其中男性患者平均58.83岁，女性患者平均62.60岁。文化程度：小学及以下114例（42.4%），初中110例（40.9%），高中及以上36例（13.4%），缺失9例（3.3%）。

### 2.2 癌症疼痛、转移及抗肿瘤治疗

治疗前无痛1例（0.4%），轻痛11例（4.1%），中痛23例（8.5%），重痛233例（86.6%），缺失1例（0.4%）；治疗后无痛7例（2.6%），轻痛174例（64.7%），中痛26例（9.7%），重痛49例（18.2%），缺失13例（4.8%），治疗前后差异有统计学意义（*Z*=-12.106, *P* < 0.001）。无肿瘤转移19例（7.1%），有转移250例（92.9%）。进行过手术治疗49例（18.2%），化疗94例（34.9%），放疗57例（21.2%）。

### 2.3 身体一般状况

KPS平均得分37.96分，无0分、80分和100分者，70分和90分各有1例，50分以下204例（75.8%），≥50分65例（24.2%）。

### 2.4 经济因素

家庭人均月收入 < 300元120例（44.6%），300元-600元50例（18.6%），≥600元58例（21.6%），缺失41例（15.2%）。实花医药费从0到10万多元不等，中位数为751.2元， < 1, 000元147例（54.6%），≥1, 000元110例（40.9%），缺失12例（4.5%）。

### 2.5 生存状况

生活质量总评分为16分-47分，平均28.76分。生活质量各条目得分情况见表 1。生活质量为极差20例（7.4%），差161例（59.9%），一般67例（24.9%），较好14例（5.2%），缺失7例（2.6%）。生存分析：随访结果有完全事件232例（86.2%），删失事件37例（13.8%）。全部因所患肿瘤死亡病例的中位生存期为10个月。1年累计生存率为43%，2年累计生存率为17%，5年累计生存率为5%，10年累计生存率 < 1%。生存函数见图 1。

**1:**
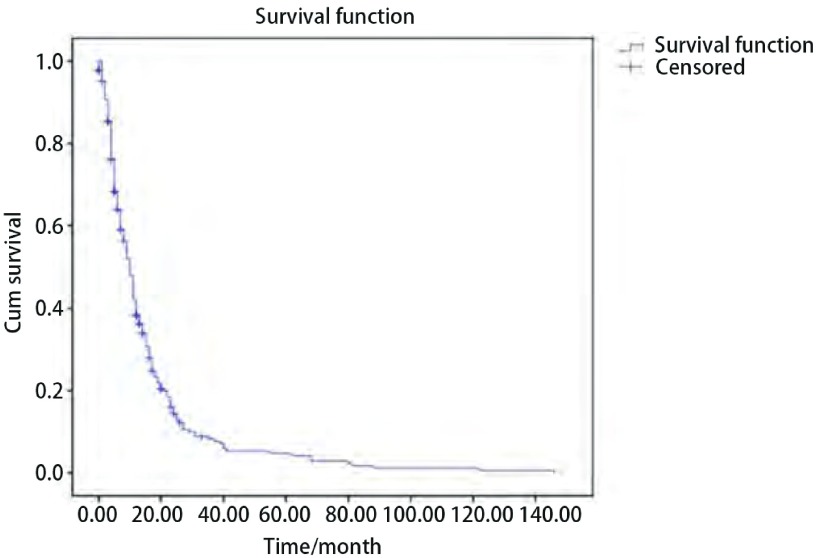
晚期肺癌死亡患者生存函数图 Survival function diagram for patients died from lung cancer

**1:** 肺癌患者生活质量各条目得分情况 The items scores for QOL of lung cancer patients

Items	Score			The inferior cases of QOL	
	Mean	SD		Cases	Proportion (%)
Orexia	2.21	0.906		204	76.7
Spirit	2.36	0.947		152	57.1
Sleep	2.61	0.962		128	48.1
Fatigue	1.82	0.720		237	89.1
Pain	2.38	0.816		153	57.5
Understanding and cooperation of family	3.65	1.026		24	9.0
Understanding and cooperation of fellows	2.15	1.341		155	58.3
Understanding of the disease	2.69	0.892		120	45.1
The attitude of treatment	2.45	1.064		157	59.0
Daily life	1.84	0.854		210	78.9
Side effects of treatment	1.89	0.796		221	83.1
Countenance	2.70	0.996		113	43.1
QOL: quality of life.					

### 2.6 生存状况的影响因素分析

生活质量评分与KPS评分呈正相关（*r*=0.446, *P* < 0.001）。生活质量评分与初始疼痛评分呈负相关（*r*=-0.259, *P* < 0.001）。由于生活质量极差和较好的患者相对较少，所以先将生活质量极差和差的合并为一组（差=1）；生活质量一般和较好的合并为一组（不差=2）然后再研究各分类变量对其的影响。单因素分析显示患者对病情的了解情况、疼痛性质、疼痛的时长、止痛效果、不良反应和营养状况对生活质量影响有统计学意义（表 2）。此外，诊疗引起的疼痛和嗜睡对生活质量的影响也有统计学意义（χ^2^=10.009, *P*=0.002; χ^2^=11.105, *P*=0.001）；而家庭人均月收入对生活质量影响无统计学意义（χ^2^=0.178, *P*=0.915）。以生活质量为因变量，单因素分析有意义的因素为自变量进行多因素*Logistic*回归分析，最终进入模型有意义的变量为患者对病情的了解情况、止痛治疗效果、KPS评分和营养状况（表 3）。生存期的单因素分析显示KPS、芬太尼使用情况、止痛效果、疼痛出现时间、医药费、放疗和手术治疗的影响有统计学意义（表 4），而家庭人均月收入的影响无统计学意义（χ^2^=1.091, *P*=0.580）。将生存期作为因变量，将对生存期影响有统计学意义的因素作为自变量代入到*Cox*模型中进行多因素生存回归分析，进入模型有意义的变量为KPS、止痛效果、疼痛出现时间和手术治疗（表 5）。

**2:** 影响肺癌患者生活质量的单因素分析 Impact analysis of every single factors on the QOL for patients with lung cancer

Influencing factors	Classes and assignment	Cases^*^	Form for QOL (%)		*χ*^2^	*P*
			Not poor	Poor		
Know illness	Adequate acquaintance=1	105	20.0	80.0	28.813	< 0.001
	Sciolism=2	85	24.7	75.3		
	Completely not clear=3	49	61.2	38.8		
The quality of pain	Knifelike pain=1	29	20.7	79.3	25.342	< 0.001
	Blunt stuffy pain=2	82	13.4	86.6		
	Other pain=3	123	45.5	54.5		
Pain length of time	Some time=1	19	63.2	36.8	16.739	< 0.001
	All the time=2	171	20.5	79.5		
Analgesic effect	Poor=1	41	29.3	70.7	9.867	0.007
	General=2	144	24.3	75.7		
	Satisfaction=3	68	45.6	54.4		
Adverse reaction	Without=1	70	44.3	55.7	7.988	0.005
	Have=2	185	25.9	74.1		
Nutritional status	Not malnutrition=1	112	40.2	59.8	10.010	0.002
	Malnutrition=2	138	21.7	78.3		
*The cases in the tab are effective which have gotten rid of the missing system.						

**3:** 肺癌患者生活质量的多因素分析结果 The results of multivariate analysis on the QOL for the patients with lung cancer

Influencing factors	B	SE	Wald	P	Exp(B)	95%CI for Exp(B)	
						Lower	Upper
Know illness	-	-	8.324	0.016	-	-	-
Know illness (1)	-2.300	0.842	7.455	0.006	0.100	0.019	0.523
Know illness (2)	-2.000	0.797	6.297	0.012	0.135	0.028	0.645
Analgesic effect	-	-	7.183	0.028	-	-	-
Analgesic effect (1)	-2.678	1.078	6.171	0.013	0.069	0.008	0.568
Analgesic effect (2)	-1.227	0.654	3.515	0.061	0.293	0.081	1.057
KPS	0.209	0.049	18.040	< 0.001	1.233	1.119	1.358
Nutritional status	-2.233	0.671	11.070	0.001	0.107	0.029	0.399
Constant	-1.752	2.361	0.551	0.458	0.173	-	-
When polytomous variable are compared use the last class as reference. Variable assignment is the same as Tab 2. KPS: Karnofsky performance status.							

**4:** 影响肺癌患者生存期的单因素分析 The influences of single factor on life cycle for patients with lung cancer

Influencing factors	Classes and assignment	Median survival (month)	*Log-rank*	*P*
KPS	≥50=1	13	9.676	0.002
	< 50=2	9		
Fentanyl used	Without=1	10	4.254	0.039
	Have=2	7		
Analgesic effect	Poor=1	7	6.946	0.031
	General=2	9		
	Satisfaction=3	13		
Radiation treatment	Without=1	9	5.193	0.023
	Have=2	14		
Surgery	Without=1	9	14.253	< 0.001
	Have=2	16		
Medical expenses	< 1, 000 RMB=1	7	8.172	0.004
	≥1, 000 RMB=2	11		
Time of pain appear	< 6 months=1	8	11.380	0.001
	≥6 months=2	15		
KPS: Karnofsky performance status.				

**5:** 多因素对肺癌患者生存期影响的*Cox*回归分析 *Cox* regression analysis for multivariate's influence of patients with lung cancer surial

Influencing factors	B	SE	Wald	*P*	Exp(B)	95%CI for Exp(B)	
						Lower	Upper
Surgery	-0.610	0.199	9.374	0.002	0.543	0.368	0.803
Time of pain appear	-0.755	0.180	17.561	< 0.001	0.470	0.330	0.669
Analgesic effect	-	-	7.087	0.022	-	-	-
Analgesic effect (1)	0.490	0.244	4.027	0.045	1.632	1.011	2.633
Analgesic effect (2)	0.466	0.183	6.502	0.011	1.594	1.114	2.281
KPS	0.641	0.202	10.101	0.001	1.898	1.279	2.819
When polytomous variable are compared use the last class as reference. Variable assignment is the same as Tab 4.							

## 3 讨论

生存时间是确定肺癌治疗投入、确定临终期和衡量治疗效果及预后的关键指标，对肺癌的临床防治有重要的指导价值，因而一直受到重视。随着医学模式的转变，生活质量这一包含患者一般健康状况、心理状态、社会生活状态及对生活满意程度的综合指标在恶性肿瘤防治评价中的应用也越来越广泛^[8]^，已成为癌症患者一个重要的观察终点和高效预后的基本组成部分^[9]^。如何使患者长期生存和提高其生活质量是目前的热门课题，本研究显示晚期肺癌患者主要集中在60岁左右的老年人且基本都发生了肿瘤的转移；治疗前晚期肿瘤患者普遍都存在重度的疼痛，治疗后疼痛缓解有统计学差异，但觉得止痛效果“一般”的患者占大多数，说明疼痛缓解有限，止痛的效果还不够理想，相关文献^[10]^也有类似结论；大部分患者一般状况较差，需要别人更多的帮助，生活自理困难，并经常需要医疗护理。

从生活质量各条目看，晚期肺癌患者感到疲乏和日常生活困难的比重较大，食欲差的比重也较大，加之癌症为消耗性疾病，所以其营养不良者的比重较大；治疗副作用的影响较严重，提示应该改善和加强治疗与护理的干预。多因素分析表明，患者对病情的了解情况、止痛治疗效果和营养状况分别为肺癌患者生活质量的独立危险因素，提示适当隐瞒患者病情、善意的谎言对于减轻患者思想压力、保证其生活质量具有重要性；有效的疼痛控制是肺癌患者生活质量的必要保证；饮食指导为患者提供抗癌食物和丰富的营养有利于提高其生活质量；此外，KPS评分与生活质量评价是一致的，良好的一般状况和自理能力、充沛的精力与好的生活质量密不可分。疼痛从多方面严重影响着肺癌患者的生活质量，除了止痛效果外，疼痛的性质、时长、程度都是很重要的影响因素，诊疗引起的疼痛对生活质量的影响提示要改良诊疗手段和设备，医护人员在服务时要手法轻柔、温馨关怀，进而减少患者的痛苦。治疗的副作用不容忽视，文献^[11]^指出便秘影响肺癌患者生活质量；本研究显示嗜睡影响生活质量，相关研究^[12]^也报道肺癌患者睡眠和生物节律紊乱与生活质量的相互关系。无论在荷兰还是日本，化疗对肺癌患者的生活质量影响明显^[11]^，本研究显示化疗对生活质量的影响无统计学意义，可能是人群及样本不同导致，也可能是化疗产生一定的痛苦和副作用而将其效果削弱。研究^[4]^指出年轻、男性、手术治疗组患者的生活质量较好，低文化程度及低收入患者的生活质量较差，可能样本各因素的具体分布情况与前者没有可比性，所以这些因素在本研究中均无统计学意义，但不可忽视文化程度对生活质量的影响，相关研究^[13]^指出文化程度相对比较低的患者心理承受能力低，遵医行为少以致生活质量低，需要医护人员多关心较低文化程度患者的心理状态。

本研究晚期肺癌患者的中位生存期仅有10个月，5年生存率基本可以忽略，这与相关文献描述一致^[14]^。多因素分析显示其独立的影响因素为有无手术治疗、出现疼痛时间、止痛治疗效果和KPS评分。正如相关文献^[15]^描述手术治疗肺癌患者生存期较长，可能与手术患者病期相对较早、体质相对较好有关，本研究中大部分患者已经无法手术，进行过手术治疗的患者仅有49例（18.9%）。出现疼痛的时间越早，越有利于早发现、早诊疗疾病，也就为延长患者生存时间创造了条件，所以提高肺癌患者生存率的关键在于早期诊断与尽可能手术治疗。文献^[16]^显示KPS评分与生存期密切相关，KPS < 50分的患者生存期明显较短。虽然本文中生活质量对生存期的影响无统计学意义，但是两者均与KPS和疼痛有相关性，我们考虑两者之间存在一定的联系。目前生活质量对生存期的影响研究还不够充分，不过有文献^[17, 18]^指出生活质量每提高10%就可能带来生存期9%的延长，且QOL可能是生存期的预测指标之一。不过，也有文献^[19]^指出生活质量中的疼痛、饮食及语言评分与生存率有关，而其它因素与生存率无关，正如本研究中生活质量总评分与生存期无关，只有其中“日常生活”一项对生存期影响有意义（χ^2^=6.011, *P*=0.014），可能因为这项与KPS评分相似，生活质量与生存期间的复杂关系还待深入研究。除上述因素外，放疗、医药费用和芬太尼的使用在单因素分析中显示出统计学意义，同样要引起注意。对于无法手术的患者，放射治疗是重要的综合治疗手段，其对提高生活质量和延长生存期可能有一定作用，其方法也正在逐步改良，以更好地减少副作用和增强治疗效果。化疗在本研究中对生存期的影响并未显现，目前对于放化疗的治疗效果研究尚存争议^[20, 21]^，不过作为重要的治疗手段在晚期肺癌治疗中仍是普遍应用的。对于贫困肿瘤患者，医药费负担很重，需要医疗政策的扶持和保险福利的帮助。芬太尼作为强效镇痛药可能是通过止痛而对生存期产生作用，并且其往往在手术中会用到，所以还有可能是通过手术治疗而起作用。另外，有文献^[22]^提到有无肿瘤转移对生存期有影响，在本研究中并未显现，可能与样本中肿瘤转移的普遍性有关；并且有无转移可能与发现的早晚和手术等有关，真正发挥作用的可能是这些因素，而且也有报道生存期与转移情况没有相关性^[23]^。上述很多文献都提到年龄对生存期的影响，本研究也未显现，可能与样本的年龄分布有关；而性别对生存期的影响无统计学意义，本研究与大部分文献相符。

疼痛对肺癌患者生活质量和生存期都有很重要的影响，所以疼痛控制应该放在主要位置，当然最好的止痛方法是抗肿瘤治疗，精神和神经方面的疼痛可以采取放松疗法和按摩等手段缓解。另外，三阶梯治疗原则仍然是最经典的癌痛镇痛治疗方法，阿片类药物仍然是镇痛治疗的主体药物；国内主张应及时、足量给药，按规定时间给药比在患者疼痛时才给药效果好。通过系统、正确地治疗与护理，90%以上患者的疼痛是可以得到缓解的^[24]^。总之，要从生活质量和生存期的主要影响因素入手，加强和完善宁养服务，进行综合干预，包括心理干预以消除患者紧张、恐惧、焦虑、抑郁等不良情绪；行为干预即了解患者的爱好并组织适合的娱乐休闲活动以分散其注意力以及运动干预等；运动干预对于增强患者活动能力，改善临床症状，促进其生活质量有积极的意义^[25]^。对于生存质量和生存期要同等重视，联合干预并进行有效的临终关怀和照顾，尽可能增加患者的舒适程度，维护临终患者的尊严，以使他们安详无遗憾地度过人生最后的旅程。
